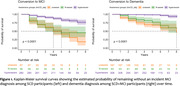# Diminished awareness, rather than hyperawareness, is more indicative for the progression along the Alzheimer’s clinical continuum, already in the subjective cognitive decline.

**DOI:** 10.1002/alz.089973

**Published:** 2025-01-03

**Authors:** Enise I Incesoy, Emrah Düzel, DELCODE study group

**Affiliations:** ^1^ German Center for Neurodegenerative Diseases (DZNE), Magdeburg Germany; ^2^ Department of Psychiatry and Psychotherapy, Otto‐von‐Guericke University, Magdeburg Germany; ^3^ Institute of Cognitive Neurology and Dementia Research (IKND), Otto‐von‐Guericke University, Magdeburg Germany

## Abstract

**Background:**

Anosognosia is a frequent phenomenon in Alzheimer’s dementia. Both heightened and decreased awareness of cognitive decline (AoCD) have been observed in mild cognitive impairment (MCI) and subjective cognitive decline (SCD). Despite some studies, the association between altered awareness and clinical conversion remained unclear. This study investigates the impact of AoCD on the conversion to incidental MCI (iMCI) and dementia within clinical high‐risk groups in the DELCODE study.

**Method:**

Annually collected longitudinal data from 405 SCD and 163 MCI participants (4.33 ±1.99 years) were used. Awareness was assessed using two scores: AoCD_inf_ (discrepancy between participant and informant reports from the SCD‐Interview) and AoCD_obj_ (discrepancy between participant‐report and dichotomized objective test scores). Participants were categorized into hyperaware (AoCD > 0), N‐aware (AoCD = 0), and unaware (AoCD < 0) groups. Cox proportional hazard regression models predicted the effect of awareness group on progression to iMCI and dementia. Analyses were adjusted for age, sex, education, MMSE, and diagnostic group.

**Results:**

*a. MCI conversion*: 109 (27%) SCD participants converted to iMCI (2.90±1.76 years). Non‐converters demonstrated more heightened initial awareness for both scores, even after adjusting for covariates (only for AoCD_obj_: F[5, 397] = 28.29, β = 0.224, p<.0001). Hyperawareness in SCD was associated with reduced risk of progression to MCI compared with diminished awareness (Hazard Ratio (HR)_inf_ = 0.57, 95% CI: 0.34‐0.98, p = .04; HR_obj_ = 0.25, 95% CI: 0.15‐0.42, p<.0001). *b. Dementia conversion*: 78 (14%) SCD+MCI participants converted to dementia (3±1.95 years). Converters exhibited significantly lower initial awareness in both scores, even after adjusting for covariates (F_obj_[5, 562] = 47.79, β = 0.231, p<.0001; F_inf_[5, 534] = 17.63, β = 0.132, p<.01). Unawareness in the nondemented clinical group was associated with higher risk of progression to dementia compared with hyperawareness (HR_inf_ = 2.26, 95% CI: 1.23‐4.15, p = .009; HR_obj_ = 3.35, 95% CI: 1.40‐8.03, p = .007).

**Conclusion:**

Unawareness of cognitive decline is related to a decreased risk of conversion to dementia in the clinical population. Specifically, in SCD, diminished awareness, rather than hyperawareness, is more indicative of progression to MCI. Informant reports and minor cognitive deficits in SCD provide valuable insights, emphasizing their importance in clinical settings and pharmacological trials.